# ZIC2 drives colorectal cancer progression by regulating QPRT-mediated cell migration

**DOI:** 10.3389/fimmu.2026.1722707

**Published:** 2026-01-29

**Authors:** Liken Zheng, Hao Liu, Ailing Yang

**Affiliations:** 1 Medical Affairs Department, Genecast Biotechnology Co., Ltd., Wuxi, Jiangsu, China; 2 College of Biotechnology and Pharmaceutical Engineering, Nanjing Tech University, Nanjing, Jiangsu, China

**Keywords:** biomarker, colorectal cancer, pan-cancer, prognosis, ZIC2

## Abstract

**Background:**

Colorectal cancer (CRC) is characterized by high mortality. Zinc finger protein ZIC2 plays a dual role in various cancers; however, its clinical value of ZIC2 in CRC remains unclear.

**Methods:**

TCGA cohort data, GEO datasets (GSE39582 and GSE139555), and a CRC_10x spatial transcriptomics dataset were used for molecular feature analysis of ZIC2 to confirm its expression and clinical significance in cancer. These analyses included bulk transcriptomic analysis, single-cell transcriptomic analysis, and spatial transcriptomic analysis. Weighted gene co-expression network analysis (WGCNA) was used to identify the genes interacting with ZIC2. Gene Ontology (GO) and Kyoto Encyclopedia of Genes and Genomes (KEGG) analyses were performed to explore the potential mechanisms of ZIC2 in CRC. Cytological assays were conducted to validate the expression, function, and mechanism of ZIC2 in CRC. The effect of ZIC2 on cell migration was evaluated using cell scratch assays.

**Results:**

ZIC2 was upregulated in most cancer tissues compared with normal tissues, and high ZIC2 expression was associated with poor prognosis. ZIC2 expression of ZIC2 was positively correlated with tumor mutational burden (TMB) and microsatellite instability (MSI). Multiple tumor-related pathways were differentially enriched between high and low ZIC2 expression phenotypes in CRC, particularly the Wnt signaling pathway and stem cell–related pathways. WGCNA identified quinolinate phosphoribosyltransferase (QPRT) as a potential downstream target regulated by ZIC2. Functional validation demonstrated that ZIC2 overexpression significantly enhanced colorectal cancer cell migration and proliferation, accompanied by upregulation of QPRT. Importantly, treatment with phthalic acid, a QPRT inhibitor, partially reversed the ZIC2-induced enhancement of cell migration, indicating that ZIC2 promotes CRC cell migration through a QPRT-dependent mechanism.

**Conclusion:**

ZIC2 may serve as a valuable prognostic and prognostic biomarker in pan-cancer, including CRC. ZIC2 may promote the colorectal cancer progression by upregulating QPRT.

## 1 Introduction

Colorectal cancer (CRC) is the third most commonly diagnosed cancer and the second leading cause of cancer-related deaths worldwide. In 2022, there were approximately 1.9 million new cases and 0.93 million deaths (1). The common risk factors include age, gender, inflammatory bowel diseases, and genetic factors (2, 3). Some progress has been made in the early diagnosis and treatment of CRC, such as the use of colonoscopy, serum biomarkers, targeted therapy, and immunotherapy (4). However, some patients with CRC are still in late stage when diagnosed at a late stage and experience rapid disease progression because of tumor heterogeneity of the CRC. Thus, it is vital to identify effective biomarkers to improve the prognosis of the patients with CRC.

The zinc finger of the cerebellum (ZIC) gene family comprises five members (ZIC1–5) that encode C2H2 zinc finger transcription factors essential for embryonic development and tissue patterning. ZIC proteins share a conserved zinc finger domain consisting of five tandem C2H2 repeats that mediate DNA binding and protein–protein interactions. These proteins regulate the development of the central nervous system, neural crest derivatives, and various organ systems, functioning as transcriptional activators or repressors depending on cellular context (5, 6). Additionally, ZIC proteins can serve as cofactors for other transcription factors, such as GLI proteins in the Hedgehog signaling pathway (7). Notably, dysregulation of ZIC family members has been implicated in various human cancers, where they function as oncogenes or tumor suppressors depending on cancer type and the cellular microenvironment.

Among the ZIC family members, Zic family member 2 (ZIC2), located on chromosome 13q32.3, has attracted particular attention in cancer research because of its context-dependent dual roles in tumorigenesis. As a transcription factor, ZIC2 can interact with DNA and protein molecules (8) and can also act as a cofactor by binding to other transcription factors (6). Initially, ZIC2 was found to play a crucial role in human growth and development, particularly in the central nervous system, where it is required for the development of neural crest derivatives, including the meningeal membrane and facial bones (9–11). In recent years, increasing evidence has demonstrated the involvement of ZIC2 in the occurrence and development of various cancers (12). However, ZIC2 exhibits dual roles in cancer. Studies have shown that the upregulated ZIC2 could induce tumor cell differentiation and inhibit cell cycle progression in breast cancer (13) and pediatric medulloblastoma (14). In contrast, ZIC2 has been identified as an oncogenic factor that promotes the tumor progression in many cancers. For example, ZIC2 promotes metastasis by activating the ERK1/2 signaling pathway in pancreatic cancer (15, 16), and ZIC2 stimulates hepatocellular carcinoma progression by regulating the expression of OCT4 and PAK4 (17, 18). In colorectal cancer, several studies have reported that ZIC2 promotes CRC development of CRC (19, 20).

The regulatory mechanisms of ZIC2 in cancer have been partially elucidated. Upstream of ZIC2, studies have reported that certain microRNAs can bind to ZIC2 and inhibit its expression, thereby impeding tumor cell growth and metastasis of tumor cells. In prostate cancer (21), nasopharyngeal carcinoma (22), and cervical cancer (23), miR-129-5p decreases ZIC2 expression of ZIC2 to reduce cell migration, invasion, and angiogenesis, and to promote apoptosis of tumor cell apoptosis. ZIC2 has also has been shown to influence tumor progression by regulating multiple downstream signaling pathways. ZIC2 can facilitate the nuclear retention via of GLI1 to enhance Hedgehog signaling and promote cell proliferation in cervical cancer (23). In prostate cancer, suppression of ZIC2 expression decreases Wnt and β-catenin expression, leading to reduced angiogenesis and epithelial–mesenchymal transition (21). Other pathways regulated by ZIC2 include the PI3K/AKT signaling pathway (24), Notch signaling pathway (25), and Src/FAK signaling pathway (26).

However, the expression pattern and functional role of ZIC2 in CRC remain incompletely understood. Therefore, this study aimed to systematically characterize ZIC2 in colorectal cancer by integrating pan-cancer analyses of ZIC2 expression and clinical outcomes using TCGA data; multidimensional transcriptomic analyses of CRC, including bulk RNA sequencing, single-cell RNA sequencing, and spatial transcriptomics; identification of ZIC2-associated regulatory targets; and experimental validation of ZIC2-mediated cell migration using wound-healing assays, as well as investigation of ZIC2-mediated transcriptional regulation of QPRT. Through this comprehensive approach, we aim to establish ZIC2 as a clinically relevant biomarker and to identify potential therapeutic targets in colorectal cancer.

## 2 Materials and methods

### 2.1 Bulk tissue transcriptomic analysis

RNA sequencing–based data of pan-cancer data from TCGA were obtained from UCSC Xena (https://xenabrowser.net/) (27), gene expression profiles were quantified using transcripts per million (TPM). Additionally, tumor mutational burden (TMB), microsatellite instability (MSI), and corresponding clinical information were also retrieved from the UCSC Xena website. Microarray data for GSE39582 were collected from the Gene Expression Omnibus (GEO) database (http://www.ncbi.nlm.nih.gov/geo/) (28). The GSE39582 dataset included 562 colorectal cancer samples.

### 2.2 Single-cell transcriptomic analysis

Single-cell RNA sequencing (scRNA-seq) data from GSE139555 (29) were downloaded from GEO. Two CRC samples and two paired normal samples were selected for further analysis. The “Seurat” (30) package was used to analyze scRNA-seq data to investigate ZIC2 expression within the colorectal cancer tumor microenvironment. Low-quality cells were excluded if the mitochondrial gene content exceeded 20%, and cells with fewer than 200 transcripts or more than 5,000 transcripts were also filtered. The single-cell data were normalized using the NormalizeData function. The *FindVariableFeatures* function was used to identify highly variable genes, and perform principal component (PCA) analysis (PCA) was performed on the top 2,000 variable genes. The top 15 principal components were used for t-distributed stochastic neighbor embedding (t-SNE). Cell clusters were annotated using the TISCH database. Cell–cell communication analysis was performed using the CellChat package (31).

### 2.3 Spatial transcriptomics analysis

Spatial transcriptomic data for primary CRC were obtained from the SpatialTME database (https://www.spatialtme.yelab.site/#!/) The CRC_10x dataset was CRC_10x, which focuses on whole-transcriptome analysis of CRC, was used in this study. Spatial cell pattern analysis was performed to visualize cell distribution and composition within CRC tissues. Spatial gene expression analysis was conducted to display the expression of ZIC2 and spatial distribution of ZIC2.

### 2.4 WGCNA analysis

Weighted correlation network analysis (WGCNA) (32) was conducted to identify genes co-expressed with ZIC2. Gene sets were obtained based on differentially expressed genes between high and low expression of ZIC2 expression groups in the TCGA-COAD dataset. A similarity matrix was constructed using the expression profiles of these genes. With a correlation coefficient threshold of 0.8, a soft-thresholding power of 3 was selected 3 as the optimal soft threshold to ensure that the network is a scale-free network topology. The similarity matrix was then transformed into an adjacency matrix, which was further converted into a topological overlap matrix (TOM). Genes were clustered into distinct modules using average linkage using hierarchical clustering based on the TOM. Module eigengenes (MEs) were defined as the first principal component of each module, and correlations between MEs and expression of ZIC2 expression were calculated to identify relevant co-expression modules which was focused on.

### 2.5 Gene function analyses

Gene Ontology (GO) and Kyoto Encyclopedia of Genes and Genomes (KEGG) enrichment analyses (33) enrichment analyses were performed using the clusterProfiler package (34). Gene sets related to proliferation, apoptosis, and invasion were obtained from the CancerSEA database (http://biocc.hrbmu.edu.cn/CancerSEA) (35). A protein–protein interaction (PPI) network was constructed using the STRING database to explore interactions between ZIC2 and other proteins. It was constructed by String database.

### 2.6 Cell culture

Human colorectal cancer cells (HCT116) were obtained from the Cell Bank of the Chinese Academy of Sciences (Shanghai, China) and cultured in RPMI 1640 medium (Gibco, Cat. No. 11875093) supplemented with 10% fetal bovine serum (Gibco, Cat. No. 10099141) and 1% penicillin–streptomycin (Gibco, Cat. No. 15140122, 100×). Cells were maintained at 37°C in a humidified atmosphere containing 5% CO_2_. Cells were routinely tested for mycoplasma contamination using the MycoAlert™ PLUS Mycoplasma Detection Kit (Lonza, Switzerland) and confirmed to be mycoplasma-free.

### 2.7 ZIC2 overexpression

HCT116 cells were transfected with either pcDNA3.1-ZIC2 (ZIC2-OE group) or an empty pcDNA3.1 vector (control group) using Lipofectamine 3000 transfection reagent (Invitrogen, Cat. No. L3000015) according to the manufacturer’s instructions. Briefly, 2 × 10^5^ cells were seeded into 6-well plates and cultured overnight. Transfection complexes were prepared by mixing 1 μg of plasmid DNA with Lipofectamine 3000 reagent in Opti-MEM medium (Gibco, Cat. No. 31985070) and incubating for 10–15 min. The complexes were then added to the cells and incubated for 48 h. Successfully transfected cells expressing high levels of ZIC2 were selected and maintained in RPMI 1640 medium containing 800 μg/mL G418 (Gibco, Cat. No. 10131035) for 2–3 weeks until stable cell lines were established. Successful ZIC2 overexpression was confirmed by Western blot analysis using an anti-ZIC2 antibody (Abcam, Cat. No. ab203625, 1:1000).

### 2.8 Western blot

Total protein from treated cells was extracted using RIPA lysis buffer (Beyotime, Cat. No. P0013B). Protein concentration was determined using the Pierce BCA Protein Assay Kit (Thermo Fisher Scientific, Cat. No. 23225). Equal amounts of protein (30–50 μg) were separated by 10% SDS-PAGE and transferred to Immobilon-P polyvinylidene fluoride (PVDF) membranes (Millipore, Cat. No. IPVH00010). Membranes were blocked with 5% non-fat milk powder in Tris-buffered saline containing 0.1% Tween-20 (TBST) for 1 h at room temperature. Primary antibodies against ZIC2 (1:1000, Abcam, Cat. No. ab203625, rabbit monoclonal), QPRT (1:1000, Abcam, Cat. No. ab234819, rabbit monoclonal), and GAPDH (1:10,000, Cell Signaling Technology, Cat. No. 2118S, rabbit monoclonal) were incubated overnight at 4 °C. After three washes with TBST, membranes were incubated with HRP-conjugated secondary antibody (goat anti-rabbit IgG, Cell Signaling Technology, Cat. No. 7074, 1:5000) for 1 h at room temperature. Protein signals were detected using enhanced chemiluminescence (ECL) reagent (Millipore, Cat. No. WBKLS0100) and visualized with a ChemiDoc imaging system (Bio-Rad).

### 2.9 CCK8 cell proliferation assay

HCT116 cells (2 × 10^4^ cells/mL) were seeded into 96-well tissue culture plates (Corning, Cat. No. 3599) at 100 μL per well in complete RPMI 1640 medium (Gibco, Cat. No. 11875093) supplemented with 10% fetal bovine serum (Gibco, Cat. No. 10099141). Cells were cultured at 37°C in a humidified atmosphere with 5% CO_2_ for 24 h to allow cell attachment. Cells from the vector control group (pcDNA3.1) and the ZIC2 overexpression group (pcDNA3.1-ZIC2) were separately seeded and cultured separately.

At designated time points (0, 24, 48, and 72 h), 10 μL of CCK8 reagent (Cell Counting Kit-8 (CCK-8; Dojindo, Cat. No. CK04-05) reagent was added to each well. Cells were incubated for an additional 2 h at 37°C in the dark. Absorbance at 450 nm was measured using a microplate spectrophotometer (Epoch, BioTek Instruments, USA), with a reference wavelength of 630 nm. All experiments were performed in triplicate with at least three independent biological replicates.

### 2.9 Wound-healing assay

HCT116 cells (1 × 10^6^) were seeded into 6-well tissue culture-treated plates (Corning, Cat. No. 3516) at a concentration of 5 × 10^5^ cells/mL in 2 mL of serum-free RPMI 1640 medium per well and incubated at 37°C with 5% CO_2_ for 24 h until reaching approximately 90% confluence was reached. A sterile 200 μL pipette tip (Eppendorf, Cat. No. 0030000850) was used to manually create three parallel scratches per well at the bottom of each well with approximately equal distances (~0.5 cm apart). Cells were then washed three times with 1× phosphate-buffered saline (PBS; Gibco, Cat. No. 10010023) to remove detached cells and cellular debris.

Scratch wounds were immediately imaged at 10× magnification 10× using a Nikon Eclipse Ti-U inverted microscope (Nikon Instruments, Japan) to record the initial wound area (0 h). Cells were then treated with phthalic acid (a QPRT inhibitor, dissolved in DMSO to a final concentration of 0.1%) at concentrations of 0 μM (control vehicle control), 10 μM, 50 μM, and 100 μM. After 24 h of treatment, wounds were re-imaged at identical positions and magnification. Migration distance was quantified using ImageJ software (version 1.53; National Institutes of Health, Bethesda, MD, USA) by measuring the wound width at 0, 24, and 48 h.

### 2.10 Dual-luciferase reporter assay

The QPRT promoter region (1.5 kb upstream of the transcription start site) was amplified by PCR and cloned into the pGL3-basic vector (Promega, Cat. No. E1741) upstream of the firefly luciferase gene. Putative ZIC2 binding sites (GCNG) were predicted using the JASPAR database. Mutant reporter constructs were generated using the QuikChange II XL Site-Directed Mutagenesis Kit (Agilent, Cat. No. 200518) and verified by DNA sequencing (BGI Genomics).

HCT116 cells seeded in 24-well plates (Corning, Cat. No. 3524) were transfected with 100 ng firefly reporter plasmid, 10 ng Renilla control plasmid (pRL-TK; Promega, Cat. No. E2241), and 500 ng pcDNA3.1 empty vector or pcDNA3.1-ZIC2 using Lipofectamine 3000 (Invitrogen, Cat. No. L3000015). Transfection complexes were prepared in Opti-MEM (Gibco, Cat. No. 31985070) for 15 min and added to cells for 6 h. Complete RPMI 1640 medium supplemented with 10% fetal bovine serum was then added, and cells were cultured for an additional 42 h.

Cells were lysed using Passive Lysis Buffer (Promega, Cat. No. E1941) and transferred to white 96-well plates (Corning, Cat. No. 3610). Luciferase activity was measured using the Dual-Luciferase Reporter Assay System (Promega, Cat. No. E1910) and a GloMax 20/20 Luminometer (Promega). Firefly luciferase activity was measured after adding 75 μL LAR II reagent was added and incubating for 2 min, followed by measurement of Renilla luciferase activity after adding 75 μL Stop & Glo reagent was added and incubating for 1 min. Relative luciferase activity was calculated as the ratio of firefly to Renilla signals and normalized to the control group. All experiments were performed in triplicate with at least three independent biological replicates.

### 2.11 Statistical analysis

All statistical analyses were performed using R software (version 4.2.1).

One-way analysis of variance (ANOVA) and Student’s t-tests were used to compare differences among groups. Kaplan–Meier survival curves and the Wilcoxon test were used for survival analysis. Pearson correlation analysis was performed to assess correlations. A p-value < 0.05 was considered statistically significant (*P < 0.05, **P < 0.01, ***P < 0.001; ns: not significant).

## 3 Results

### 3.1 Bulk analysis of expression of ZIC2 in pan cancer

**Figure 1:**
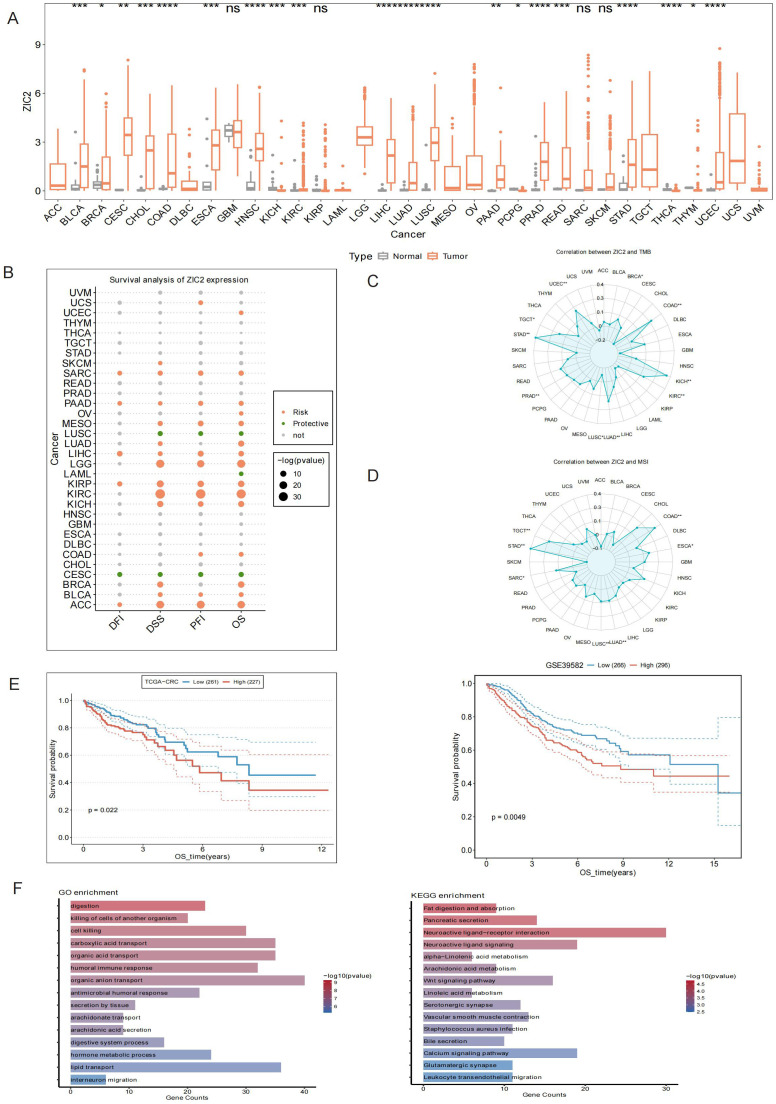
Bulk analysis of ZIC2 in pan cancer. **(A)** ZIC2 expression of ZIC2 in normal tissue and cancer tissues across pan-cancer. **(B)** Prognostic analysis of ZIC2 expression of ZIC2 in pan-cancer. **(C)** Tumor mutational burden (TMB) analysis in relation to ZIC2 expression of ZIC2 in pan-cancer. **(D)** Microsatellite instability (MSI) analysis in relation to ZIC2 expression of ZIC2 in pan-cancer. **(E)** Prognostic analysis of ZIC2 expression of ZIC2 in colorectal cancer based on TCGA-COAD and GSE39582. **(F)** Gene Ontology (GO) and Kyoto Encyclopedia of Genes and Genomes (KEGG) enrichment analyses based on ZIC2 expression of ZIC2 in CRC. *p < 0.05, **p < 0.01, ***p < 0.001, ****p <0.0001..

We first conducted a pan-cancer analysis of ZIC2 expression of ZIC2 based on TCGA data. The results showed that ZIC2 expression of ZIC2 was significantly upregulated in bladder urothelial carcinoma (BLCA), breast invasive carcinoma (BRCA), cervical squamous cell carcinoma and endocervical adenocarcinoma (CESC), cholangiocarcinoma (CHOL), colon adenocarcinoma (COAD), esophageal carcinoma (ESCA), head and neck squamous cell carcinoma (HNSC), kidney renal clear cell carcinoma (KIRC), liver hepatocellular carcinoma (LIHC), lung adenocarcinoma (LUAD), lung squamous cell carcinoma (LUSC), pancreatic adenocarcinoma (PAAD), prostate adenocarcinoma (PRAD), rectum adenocarcinoma (READ), stomach adenocarcinoma (STAD), and uterine corpus endometrial carcinoma (UCEC). In contrast, ZIC2 expression of ZIC2 was significantly downregulated in kidney chromophobe (KICH), pheochromocytoma and paraganglioma (PCPG), thyroid carcinoma (THCA), and thymoma (THYM) (**Figure 1A**).

Prognostic analysis revealed that higher ZIC2 expression was associated with shorter disease-free interval (DFI), disease-specific survival (DSS), progression-free interval (PFI), or overall survival (OS) in uterine carcinosarcoma (UCS), uterine corpus endometrial carcinoma (UCEC), skin cutaneous melanoma (SKCM), sarcoma (SARC), PAAD, ovarian serous cystadenocarcinoma (OV), mesothelioma (MESO), LUAD, LIHC, brain lower-grade glioma (LGG), kidney renal papillary cell carcinoma (KIRP), KIRC, KICH, COAD, BRCA, BLCA, and adrenocortical carcinoma (ACC). On the contrary, lower ZIC2 expression was associated with shorter DFI, DSS, PFI, or OS in LUSC, acute myeloid leukemia (LAML), and CESC (**Figure 1B**).

We further analyzed the correlation between ZIC2 expression of ZIC2 and tumor mutational burden (TMB) across pan-cancer datasets. ZIC2 expression showed a positive correlation with TMB in several cancers, including BRCA, COAD, KICH, KIRC, LUAD, PRAD, STAD, testicular germ cell tumors (TGCT), and UCEC, whereas a negative correlation was observed in LUSC (**Figure 1C**). Regarding microsatellite instability (MSI), ZIC2 expression was positively correlated with MSI status in COAD, ESCA, LUAD, LUSC, SARC, STAD, and TGCT (**Figure 1D**).

Next, we focused on the role of ZIC2 in CRC. Kaplan–Meier analysis showed that CRC patients with higher ZIC2 expression had significantly shorter OS (P = 0.022), and the prognostic analysis in CRC patients from the GSE39582 dataset showed a consistent trend (**Figure 1E**). Gene Ontology (GO) analysis based on TCGA-COAD data indicated that ZIC2 was involved in 170 processes, including 93 biological processes, 14 cellular components, and 63 molecular functions. The top enriched terms included digestion, killing of cells of another organism, and cell killing. Kyoto Encyclopedia of Genes and Genomes (KEGG) enrichment analysis identified 12 pathways are significantly enriched pathways, such as fat digestion and absorption, pancreatic secretion, and neuroactive ligand–receptor interaction (**Figure 1F**).

To further explore the clinical significance of ZIC2 in CRC, we analyzed the correlation between ZIC2 expression and clinicopathological parameters in the TCGA-COAD cohort. ZIC2 expression showed a statistically significant associations with patient age and gender (**Supplementary Figures S1A, B**). However, no significant correlations were observed between ZIC2 expression and pathological TNM stage parameters (**Supplementary Figures S1C–F**). These results suggest that while ZIC2 expression is associated with demographic factors and may represent an independent prognostic marker distinct from conventional TNM staging in CRC patients.

### 3.2 Single cell RNA-sequencing analysis of expression of ZIC2 expression of ZIC2 in CRC

**Figure 2:**
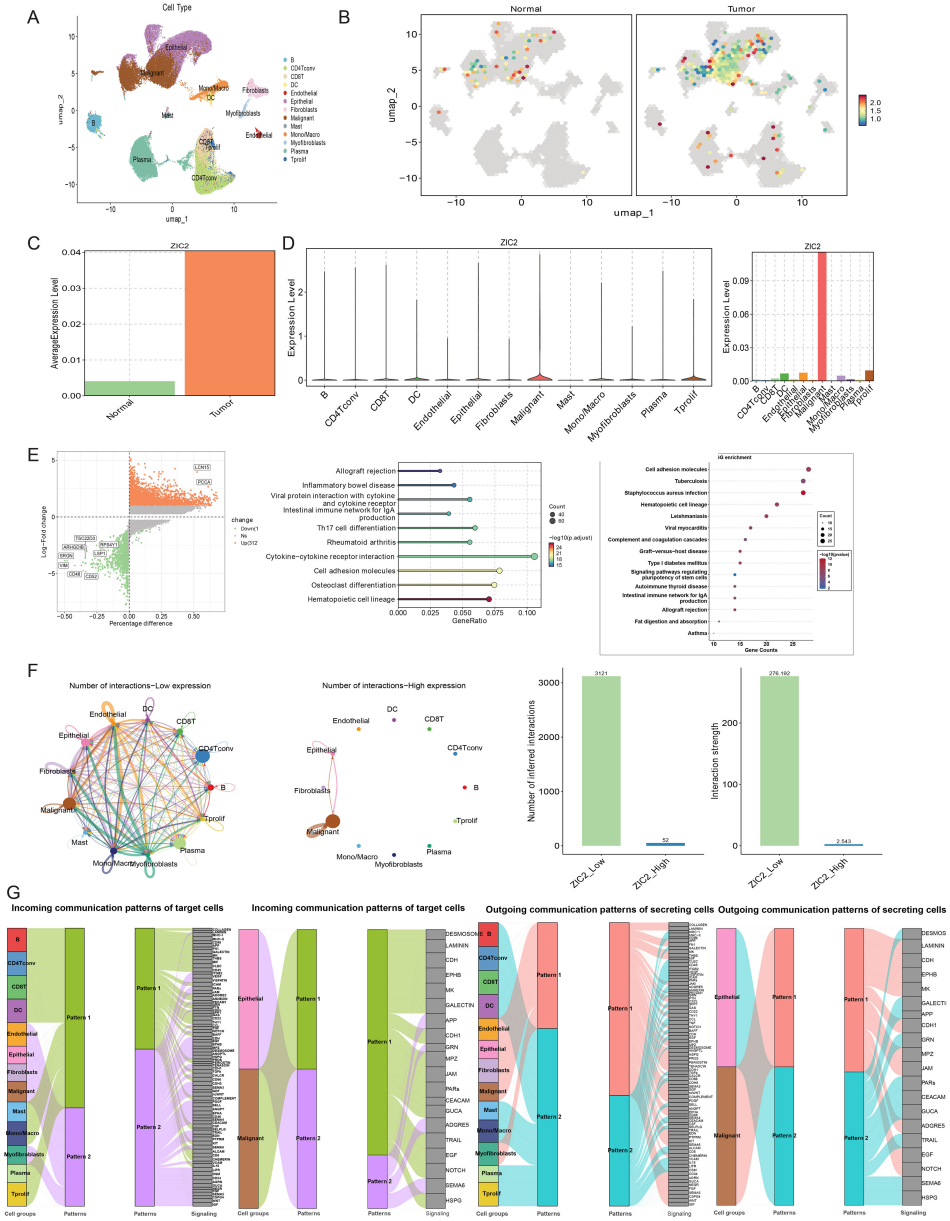
scRNA-seq analysis. **(A)** Cell-type annotation visualized by UMAP in CRC tissues. **(B)** Distribution of ZIC2 expression across different cell types. **(C)** Quantitative analysis of ZIC2 expression of ZIC2 in CRC tissues compared with normal tissues. **(D)** Quantitative analysis of ZIC2 expression of ZIC2 in CRC tissues. **(E)** Differentially expressed genes identified in cancer tissues based on ZIC2 expression. **(F)** Cell–cell communication analysis in CRC. **(G)** Incoming and outgoing communication patterns in high and low ZIC2 expression of ZIC2 groups.

To explore the distribution of ZIC2 expression within the tumor microenvironment, we performed single-cell RNA sequencing (scRNA-seq) analysis using the GSE139555 dataset. After quality control, thirteen cell types of cells were identified in CRC tissues, including B cells, CD4 conventional T cells, CD8 T cells, dendritic cells, endothelial cells, epithelial cells, fibroblasts, malignant cells, mast cells, monocytes/macrophages, myofibroblasts, plasma cells, and proliferating T prolif cells (**Figure 2A**).

In normal tissue, ZIC2 mainly expressed in epithelial cell, and in cancer tissue, ZIC2 mainly expressed in epithelial cell and malignant cell, it also expressed in mono/macro cell, CD4 and CD8 T cell (**Figures 2B, D**), besides, it also showed that the ZIC2 was up-regulated in tumor tissue than normal tissue (**Figure 2C**). According to the expression of ZIC2, we divided the cells in cancer tissue into two groups, high expression of ZIC2 group and low expression of ZIC2 group. Differentially expressed genes between these two groups were identified (**Figure 2D**), with 1,606 downregulated genes down-regulated and 3,127 upregulated genes. KEGG enrichment analysis revealed enrichment of multiple pathways, including cell adhesion molecule pathways and stem cell–related pathways (**Figure 2E**). Cell–cell communication analysis showed that the number of interactions in the low ZIC2 expression of ZIC2 group was greater than that of in the high ZIC2 expression of ZIC2 group (**Figures 2F, G**). In the high ZIC2 expression of ZIC2 group, the cell–cell communication mainly occurred between malignant cells and endothelial cells.

### 3.3 Spatial transcriptomics analysis of ZIC2 in CRC

**Figure 3:**
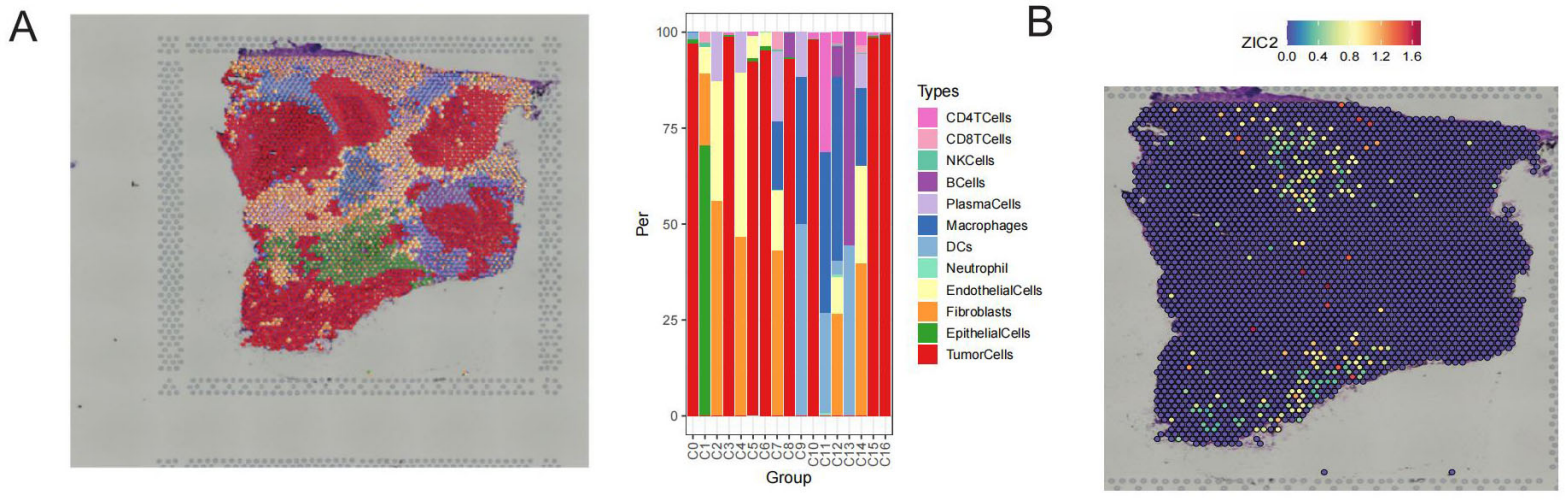
Spatial transcriptomics analysis of ZIC2 in CRC. **(A)** Cellular composition and spatial distribution of cells in CRC tissues. **(B)** Spatial distribution of ZIC2 expression of ZIC2.

We further explored the relationship between ZIC2 expression and spatial distribution in CRC using spatial transcriptomics analysis. The cellular composition and spatial distribution of cells were visualized in **Figure 3A**, in which twelve cell types were annotated, including tumor cells, fibroblasts, and others. ZIC2 expression was predominantly localized in tumor cells (**Figure 3B**).

### 3.4 Identification of the genes interacting with ZIC2

**Figure 4:**
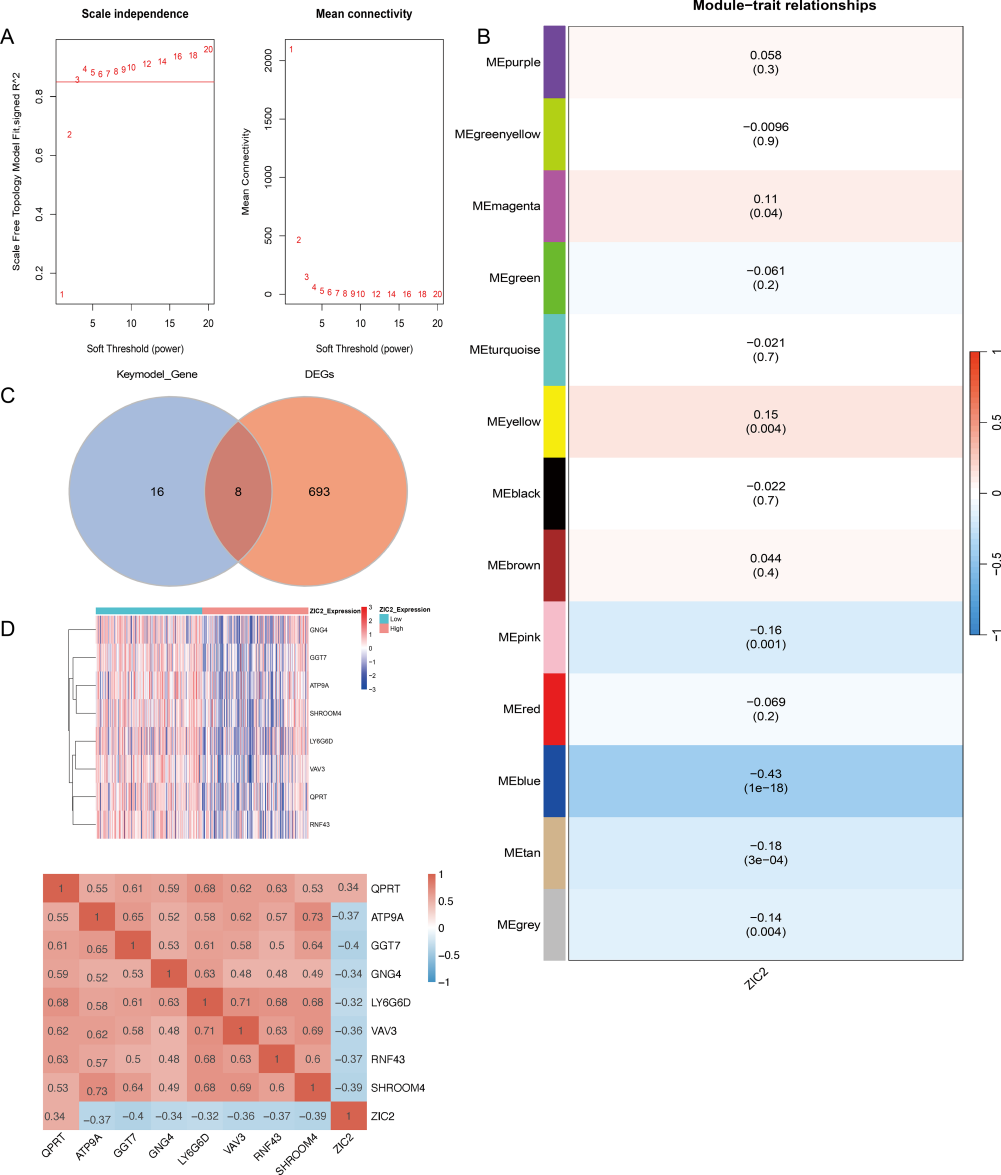
Function analysis of ZIC2 in CRC. **(A)** Optimal soft threshold; **(B)** Clustering heatmap of WGCNA; **(C)** Venn diagram for screening genes related to ZIC2; **(D)** Transcription factor analysis for filtered genes.

To explore the function and potential mechanism of ZIC2 in CRC. First, we conducted weighted gene co-expression network analysis (WGCNA) to identify the co-expression genes associated with ZIC2. The results showed that multiple modules were significant when the optimal soft-thresholding power was 3 (**Figures 4A, B**). The blue module was selected for further analysis, as it showed a strong association based on module membership (>0.7) and gene significance (>0.3). A total of 24 genes were identified from this module. We then filtered the genes that were differentially expressed between the high ZIC2 expression of ZIC2 group and the low ZIC2 expression of ZIC2 group in this dataset and identified 701 differentially expressed genes. By intersecting these two gene sets, we found that eight genes overlapped between the two groups, including QPRT (**Figure 4C**). QPRT was upregulated in cancer tissues compared with normal tissues, and its expression of QPRT was positively correlated with ZIC2 expression (**Figure 4D**).

### 3.5 ZIC2 promotes CRC cell migration by upregulating QPRT expression of QPRT and activating Wnt pathway

**Figure 5:**
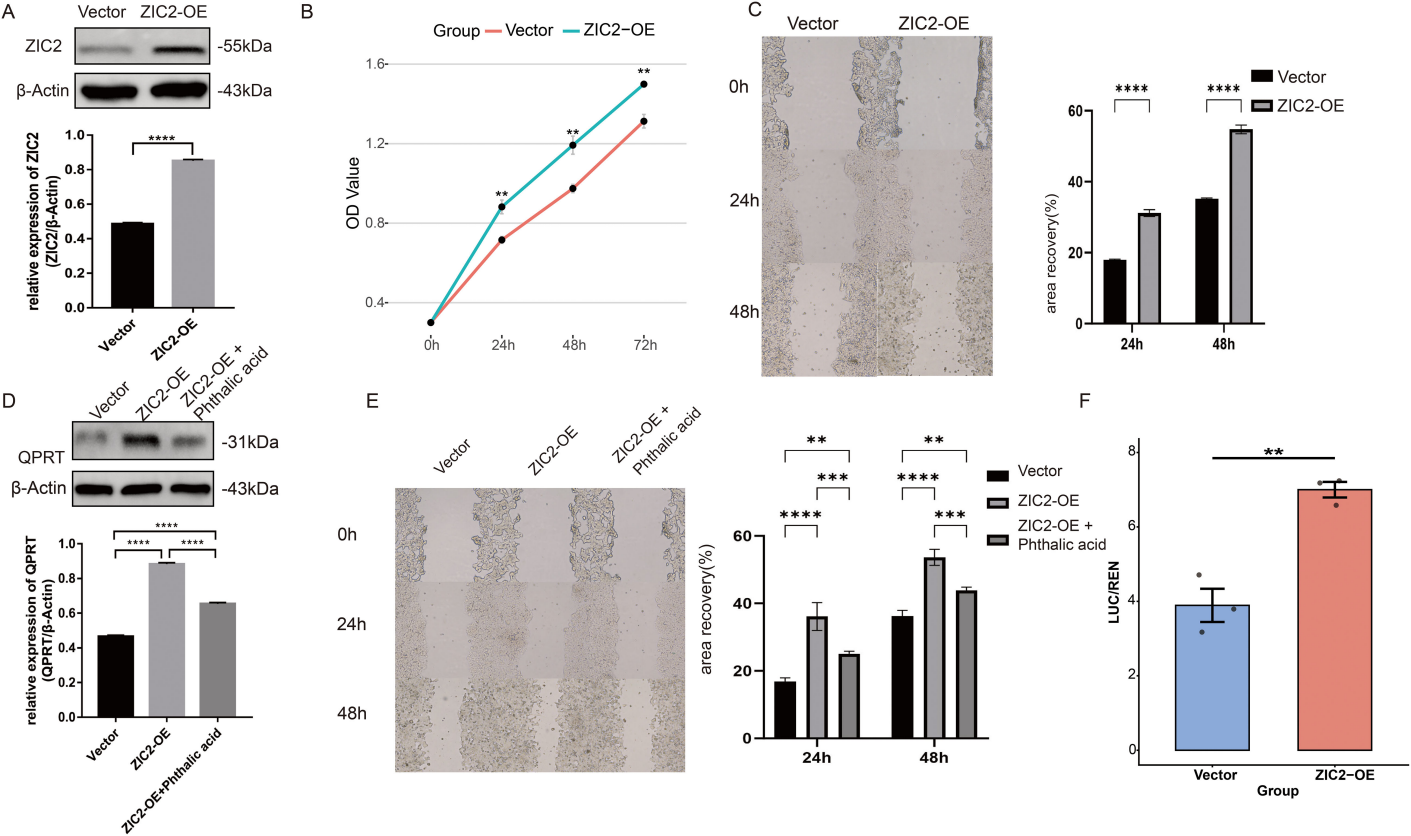
Function verification of ZIC2 via arrays. **(A)** Western blot analysis confirming construction of CRC cell lines with ZIC2 overexpression (ZIC2-OE); grayscale values were used for quantitative analysis. **(B)** CCK-8 assay comparing cell proliferation between vector control and ZIC2-OE groups. **(C)** Wound-healing assay showing migration differences between vector control and ZIC2-OE groups; migration rates were quantified. **(D)** Western blot analysis and quantification of QPRT expression in vector control, ZIC2-OE, and ZIC2-OE plus phthalic acid groups. **(E)** Wound-healing assay and quantitative analysis for the expression change of cell migration in vector control, ZIC2-OE, and ZIC2-OE plus phthalic acid groups. **(F)** Dual-luciferase reporter assay demonstrating direct binding of ZIC2 to the QPRT promoter. **P < 0.01, ***P < 0.001, ****P < 0.0001.

To further to confirm the biological role of ZIC2 in CRC cells, we first successfully constructed the CRC cell lines with ZIC2 overexpression (ZIC2-OE) (**Figure 5A**). The CCK-8 assay showed that the proliferative capacity of colorectal cancer cells was significantly enhanced following ZIC2 overexpression of ZIC2 (**Figure 5B**). Wound-healing assays demonstrated that cell migration was markedly increased in the ZIC2 overexpression group compared with the vector control group (**Figure 5C**).

Phthalic acid was used as an inhibitor of QPRT. We examined QPRT. We detected the expression of QPRT in the vector control cells, ZIC2 overexpression cells, and ZIC2 overexpression cells treated with phthalic acid. The results showed that QPRT expression of QPRT was highest in ZIC2-OE cells, reduced in ZIC2-OE cells, ZIC2-OE cells treated with phthalic acid, and lowest in vector control cells (**Figure 5D**). Consistently, wound-healing assays revealed that cell migration was fastest in the ZIC2 overexpression group, and slowest in the vector control group, while migration of cells in the ZIC2 overexpression plus phthalic acid group showed an intermediate phenotype (**Figure 5E**).

In addition, we performed a dual-luciferase reporter assay was performed to verify direct transcriptional regulation of QPRT by ZIC2. ZIC2 overexpression significantly enhanced luciferase activity driven by the QPRT promoter, indicating that ZIC2 directly binds to and activates the QPRT promoter (**Figure 5F**).

## 4 Discussion

Colorectal cancer is characterized by strong invasiveness and high mortality (4). Although traditional clinicopathological parameters can provide some indication of overall survival (OS) in patients with CRC, their predictive accuracy is limited due to tumor heterogeneity. Similar limitations apply to current CRC treatments on CRC (36). Therefore, identifying specific biomarkers for predicting CRC prognosis is necessary, and such biomarkers may further serve as therapeutic targets to improve clinical outcomes in patients with CRC.

ZIC2 is a protein consisting of 532 amino acids, including an N-terminal domain involved in transcriptional regulation, five C2H2 zinc finger repeats that function as DNA-binding domains, and a C-terminal domain (37). ZIC2 was initially identified as an essential regulator of human nervous system development (37). In recent years, ZIC2 has attracted increasing attention in the field of cancer research. Numerous studies have reported that the ZIC2 is significantly associated with tumor growth and metastasis in patients, and may serve as a potential tumor marker or therapeutic target. However, the role of ZIC2 appears to be context dependent and varies across cancer types of cancers. In the present study, we systematically analyzed the characteristics of ZIC2 across pan-cancer datasets using TCGA data and we further validated the potential diagnostic and prognostic value of ZIC2 in CRC through multiple biological experiments.

First, we compared the expression of ZIC2 across 33 cancers and their corresponding normal tissues, and found that ZIC2 was differentially expressed in 20 cancers, with most showing upregulation in cancer tissues. Prognostic analyses indicated that ZIC2 expression was associated with disease-specific survival (DSS), disease-free interval (DFI), progression-free interval (PFI), and overall survival (OS). In most tumors, high ZIC2 expression was generally associated with poorer DSS, DFI, PFI, or OS. Tumor mutation burden (TMB) and microsatellite instability (MSI) are recognized as biomarkers for immunotherapy (38, 39). Therefore, we analyzed the correlations between ZIC2 expression of ZIC2 and TMB and MSI across pan-cancer datasets. We found that there was a positive associations between ZIC2 expression of ZIC2 and TMB or MSI in most cancers, suggesting that ZIC2 may participate in the tumorigenesis by influencing TMB and MSI. These findings are consistent with a previous study (37), and suggest that ZIC2 has potential as a diagnostic or prognostic biomarker in cancer. In CRC, and high ZIC2 expression of ZIC2 was associated with poor prognosis, and this result was also validated in the GSE39582 cohort. In addition, ZIC2 expression of ZIC2 was significantly associated with TMB and MSI. However, the function and underlying mechanisms of ZIC2 in CRC remain unclear; therefore, we further explored its role in CRC. Gene Ontology (GO) and Kyoto Encyclopedia of Genes and Genomes (KEGG) pathway analyses indicated that ZIC2 is involved in multiple tumor-related pathways, including cytokine–cytokine receptor interaction, the Wnt signaling pathway, and leukocyte transendothelial migration. These results provide potential mechanistic insights into the role of ZIC2 in CRC progression. Previous studies have reported that ZIC2 facilitates CRC progression and metastasis through activation of the TGF-β signaling pathway (19), Wnt signaling pathway (20) and Hedgehog signaling pathway (40).

Colorectal cancer is a complicated and multifactorial disease, and many studies have emphasized the importance of the tumor microenvironment (41, 42). We have found that ZIC2 expression was associated with tumor mutational burden (TMB) and microsatellite instability (MSI). However, limited research has focused on the role of ZIC2 within the tumor microenvironment. Thus, we further comprehensively analyzed the distribution and function of ZIC2 based on the scRNA-seq dataset GSE184198. We found that ZIC2 was expressed in multiple cell types. In normal tissues, ZIC2 was mainly expressed in epithelial cells, whereas in tumor tissues, the top three cell types expressing ZIC2 were malignant cells, proliferating T prolif cells, and epithelial cells. The expression of ZIC2 in tumor tissues was higher than that of in normal tissues, which was consistent with the results of the bulk analysis. Moreover, we analyzed the function of ZIC2 in CRC by dividing cells into two groups according to ZIC2 expression. A total of 4,733 differentially expressed genes were identified between the two groups. These genes were involved in multiple tumor-related pathways, such as cytokine–cytokine receptor interaction and cell adhesion molecules, providing clues for elucidating the role of ZIC2 in the tumor microenvironment of CRC. Interestingly, CellChat analysis showed that cell–cell interactions were more active in the high ZIC2 expression of ZIC2 group. These interactions were mainly observed among malignant cells and epithelial cells, suggesting that ZIC2 may regulate the transition from normal cells to cancer cells. Spatial transcriptomics further confirmed that ZIC2 was predominantly expressed in tumor cells.

We conducted WGCNA to identify genes interacting with ZIC2, and we found that quinolinate phosphoribosyltransferase (QPRT) may be regulated by ZIC2. QPRT is a rate-limiting enzyme involved in tryptophan degradation to nicotinamide adenine dinucleotide (NAD^+^) through the kynurenine pathway (43). Previous studies have shown that QPRT contributes to the breast cancer progression by activating PI3K/AKT signaling pathway (44, 45). Targeting QPRT could enhance cisplatin sensitivity and inhibit the PI3K/AKT signaling pathway in ovarian cancer cells (46). Our study found QPRT was upregulated in cancer tissue than that in normal tissue. Furthermore, the expression of QPRT was positively related to the expression of ZIC2. ZIC2 acts as a C2H2-type zinc finger transcription factor that can directly bind promoter regions of target genes to regulate transcription. Although QPRT was identified QPRT as a key downstream gene associated with ZIC2, the direct promoter occupancy of QPRT by ZIC2 requires further experimental validation. Mechanistically, upregulation of QPRT enhances NAD^+^ biosynthesis via the kynurenine pathway, which may support cellular processes such as energy production, metabolism, and activate signaling enzymes important for cell migration (47). The observation that QPRT inhibition can only partially reversed ZIC2-induced migration suggests that ZIC2-mediated migration likely involves additional parallel pathways.

To confirm the expression, function, and potential mechanisms of ZIC2 in CRC, we conducted a series of biological experiments. We demonstrated that ZIC2 overexpression promoted colorectal cancer cell proliferation and migration of colorectal cancer cell, and inhibited apoptosis. When we added phthalic acid was added following ZIC2 overexpression, these malignant phenotypes were partially reversed, indicating that ZIC2 may promote CRC progression through interaction with QPRT. These findings suggest that ZIC2 may represent a promising therapeutic target and prognostic biomarker for CRC.

There are several limitations in our present study. First, clinical samples need to be collected to verify the clinical value of ZIC2 for diagnosis and prognosis of CRC. Secondly, we just validated the biological function of ZIC2 in colorectal cancer. In the future, this should be further validated using animal experiments. Third, our *in vitro* validation relied solely on the HCT116 cell line; future studies should validate key findings across multiple distinct colorectal cancer cell lines (such as SW480, LoVo, or DLD-1) to exclude cell line–specific effects and ensure the generalizability of our results.

## 5 Conclusion

This study comprehensively investigated the clinical significance of ZIC2 in CRC. We found that ZIC2 may serve as a potential prognostic indicator in CRC. ZIC2 overexpression significantly promoted colorectal cancer cell proliferation and migration of colorectal cancer cell by interacting with QPRT. Our findings indicate that ZIC2 plays an oncogenic role in CRC, and represents a promising therapeutic target and prognostic biomarker.

## Data Availability

The original contributions presented in the study are included in the article/**Supplementary Material**. Further inquiries can be directed to the corresponding author.

## Supplementary material

Supplementary Figure 1 Correlation analysis between ZIC2 expression and clinicopathological parameters in the TCGA-COAD cohort. A. Age (≤65 vs >65). B. Gender (male vs female) C. Pathological T stage, D. Pathological N stage, E. Pathological M stage, F. Stage. *P < 0.05, **P < 0.01, ns: not significant.
