# Supplementary figures and images for: ZIC2 drives colorectal cancer progression by regulating QPRT-mediated cell migration
Source: Front Immunol. 2026 Jan 29;17:1722707. doi: 10.3389/fimmu.2026.1722707 (PMC12894010; doi:10.3389/fimmu.2026.1722707)

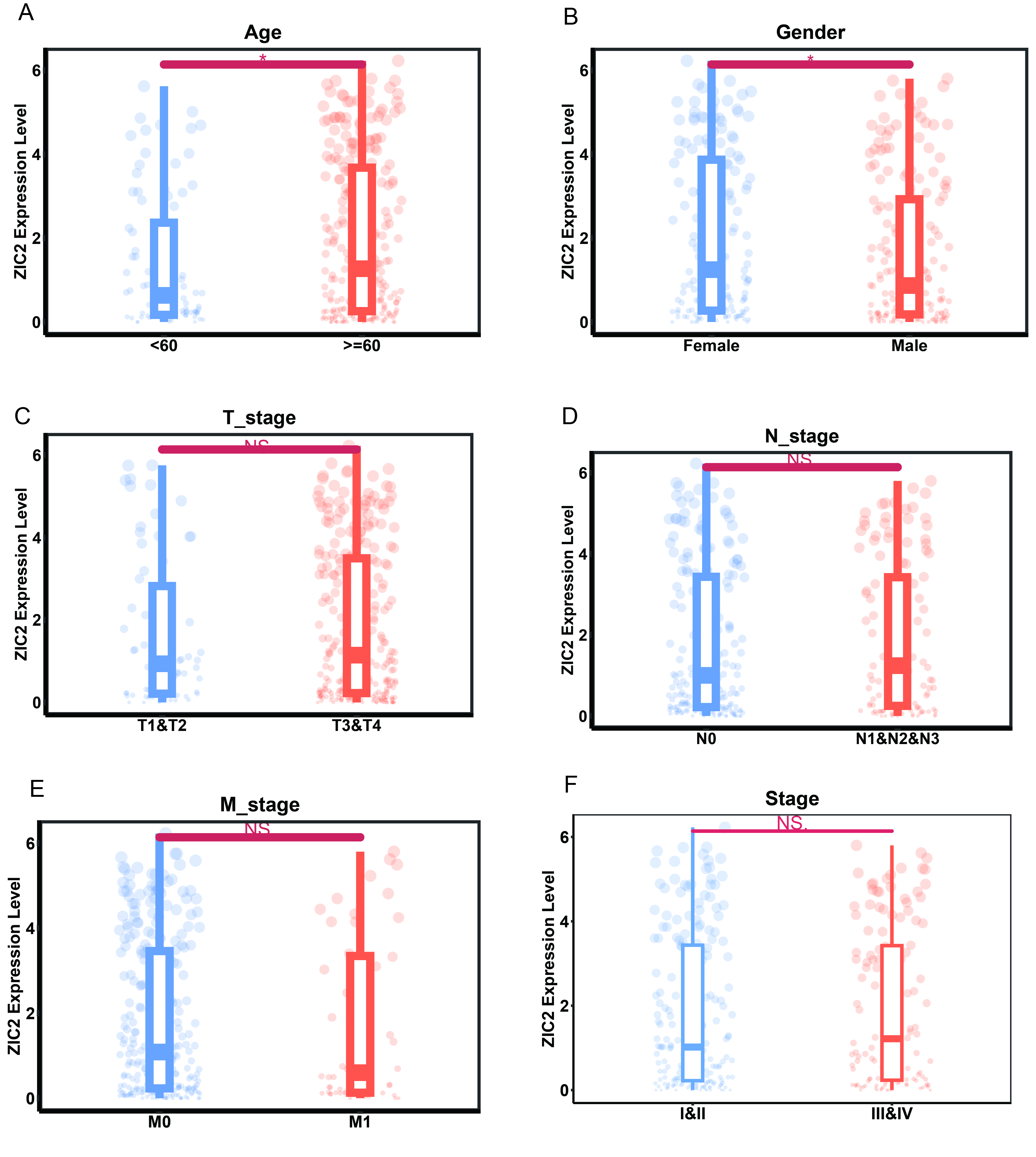

Supplement: Supplementary Figure 1 — Correlation analysis between ZIC2 expression and clinicopathological parameters in the TCGA-COAD cohort. A. Age (≤65 vs >65). B. Gender (male vs female) C. Pathological T stage, D. Pathological N stage, E. Pathological M stage, F. Stage. *P < 0.05, **P < 0.01, ns: not significant. [file Image1.jpeg]

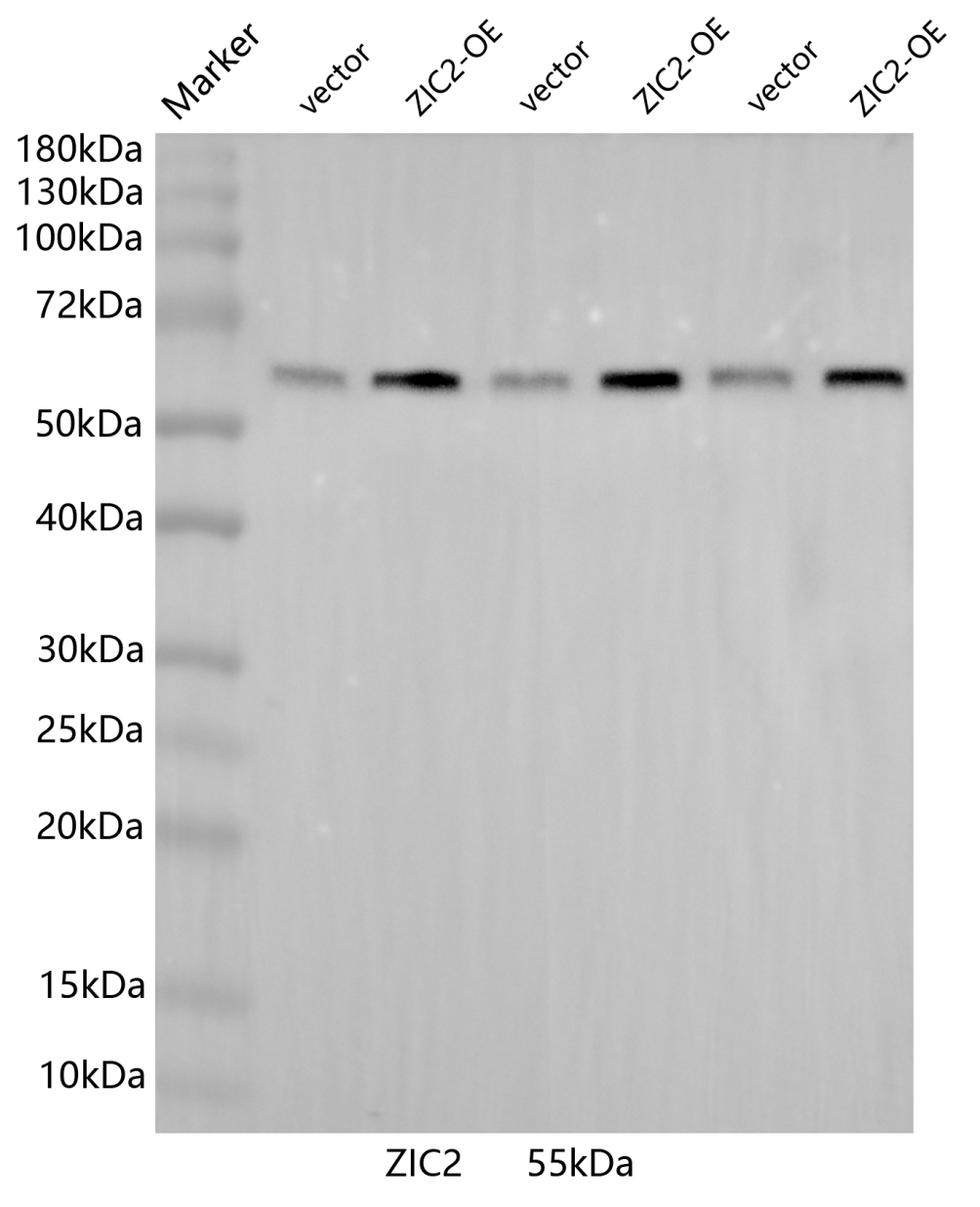


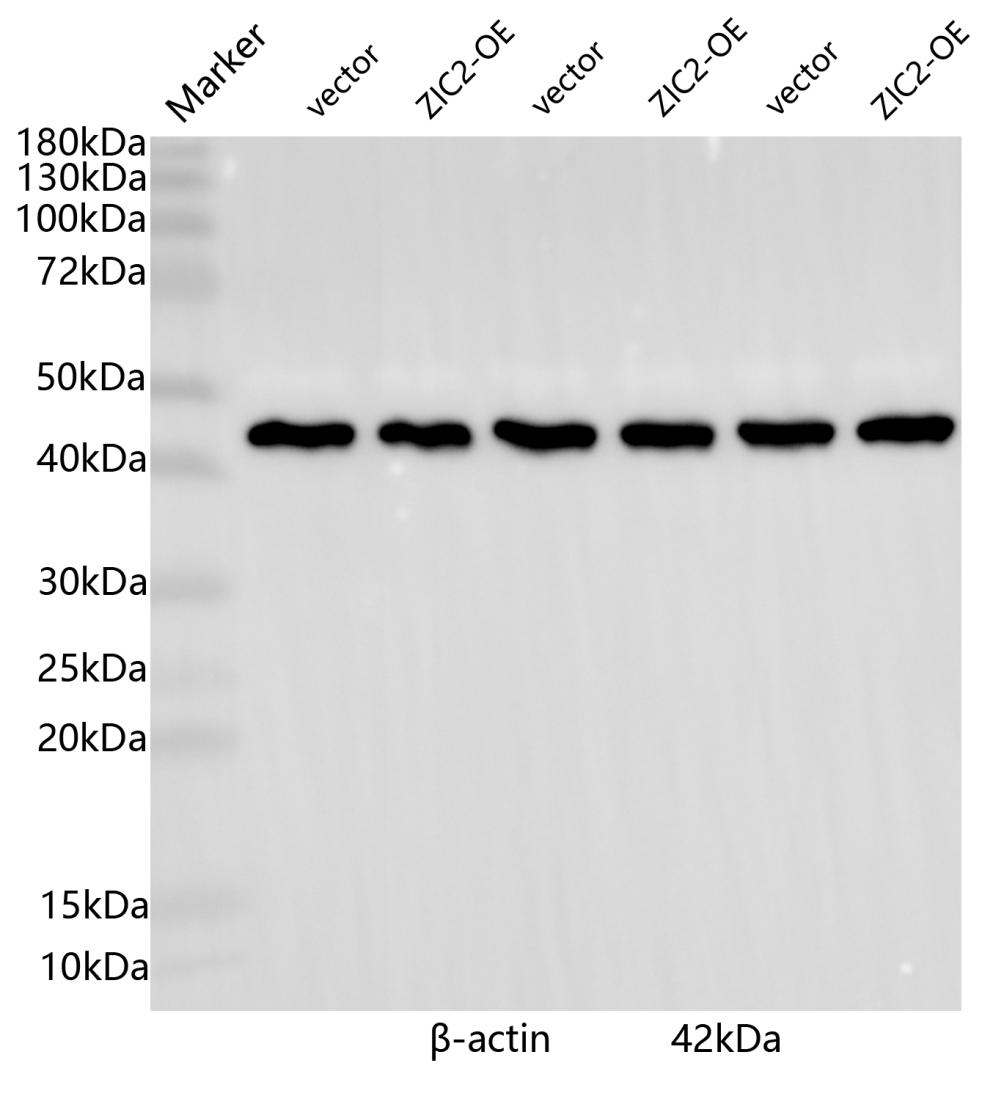


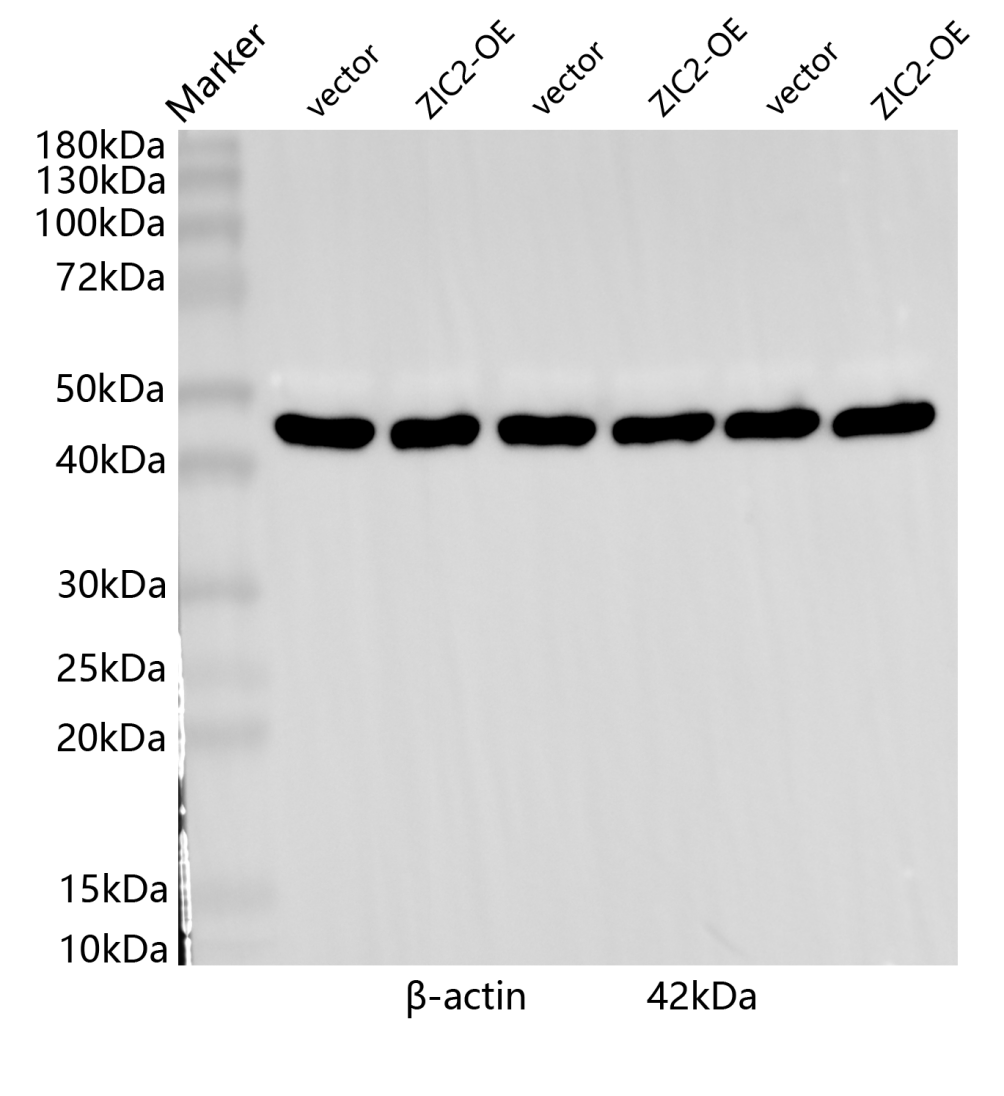


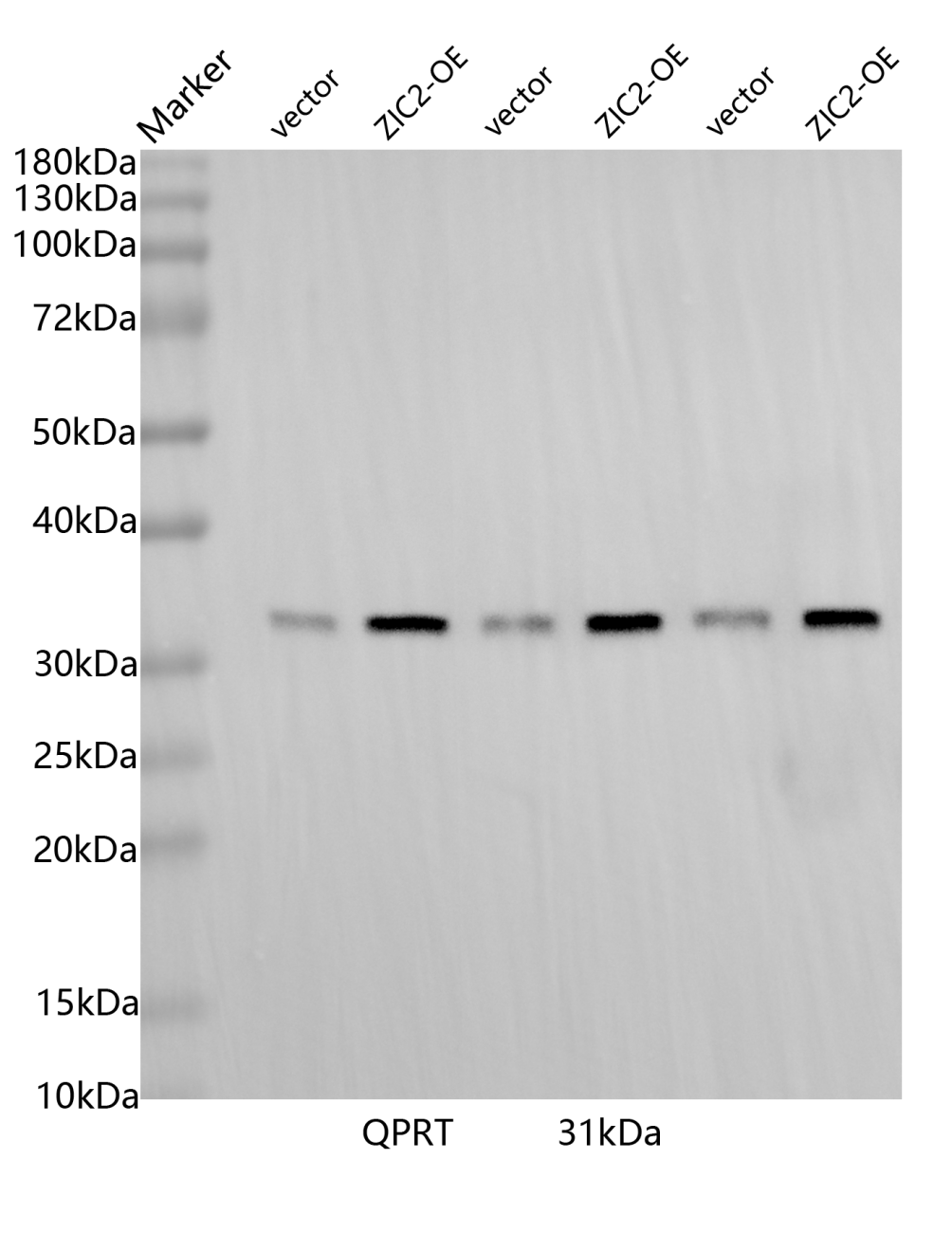


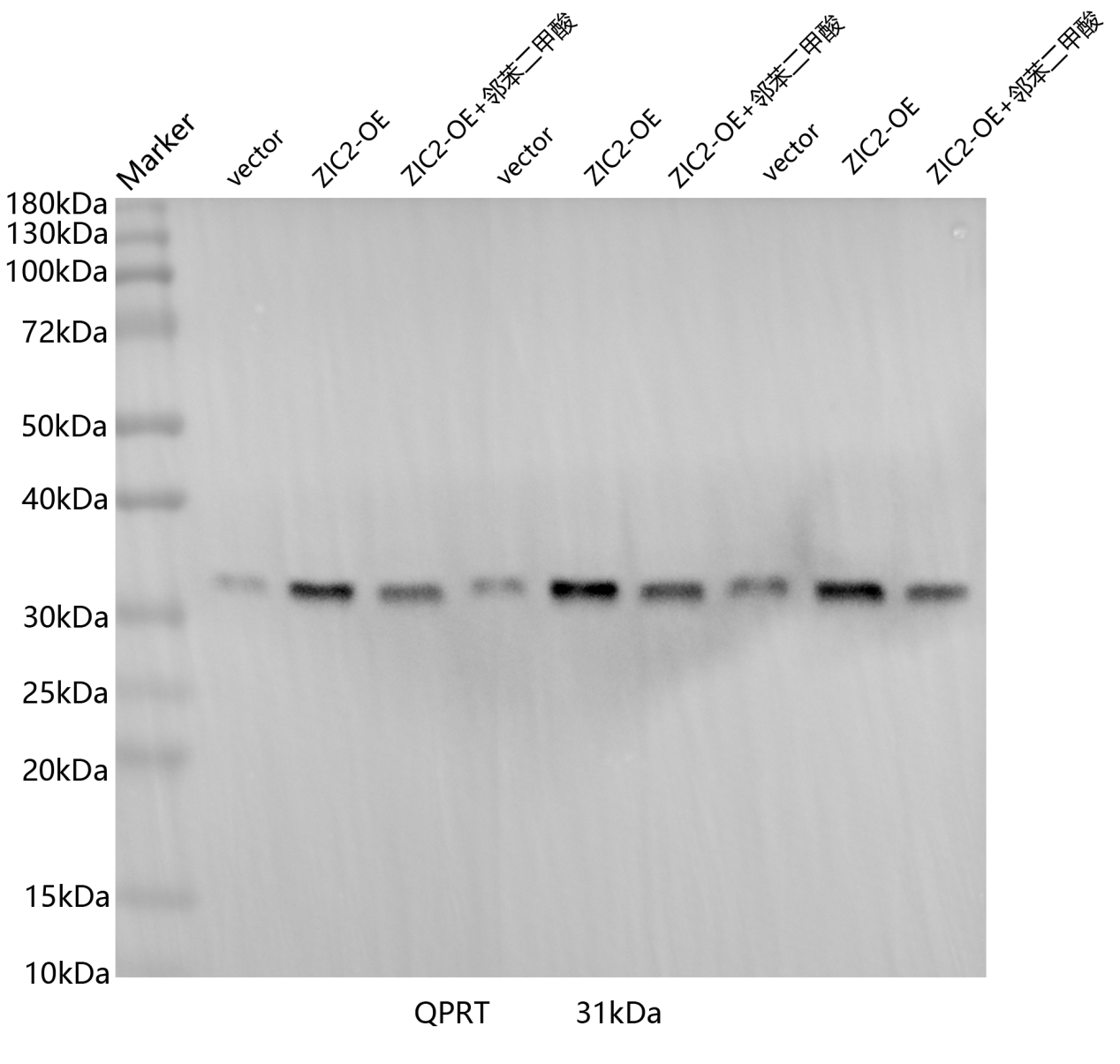


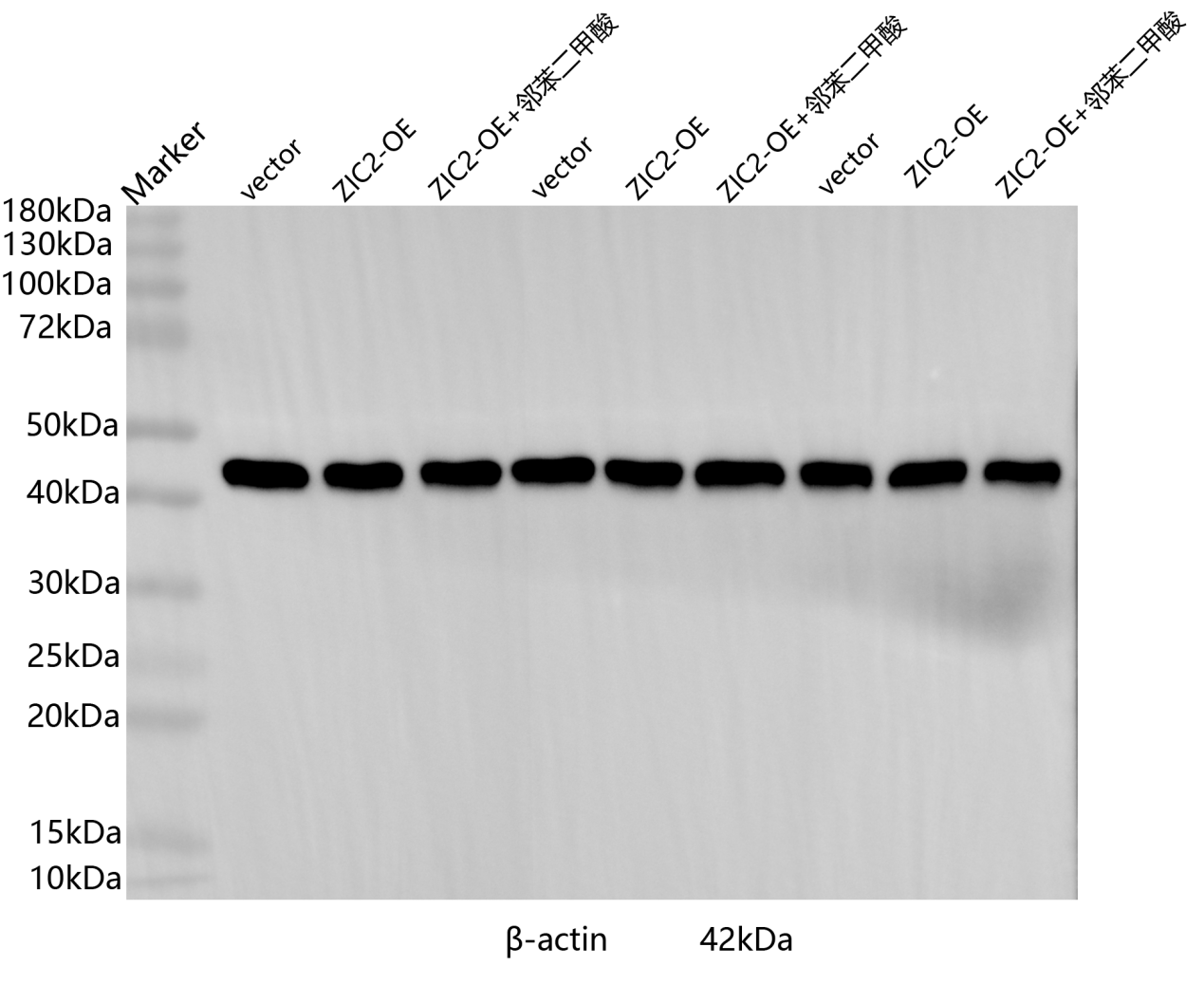


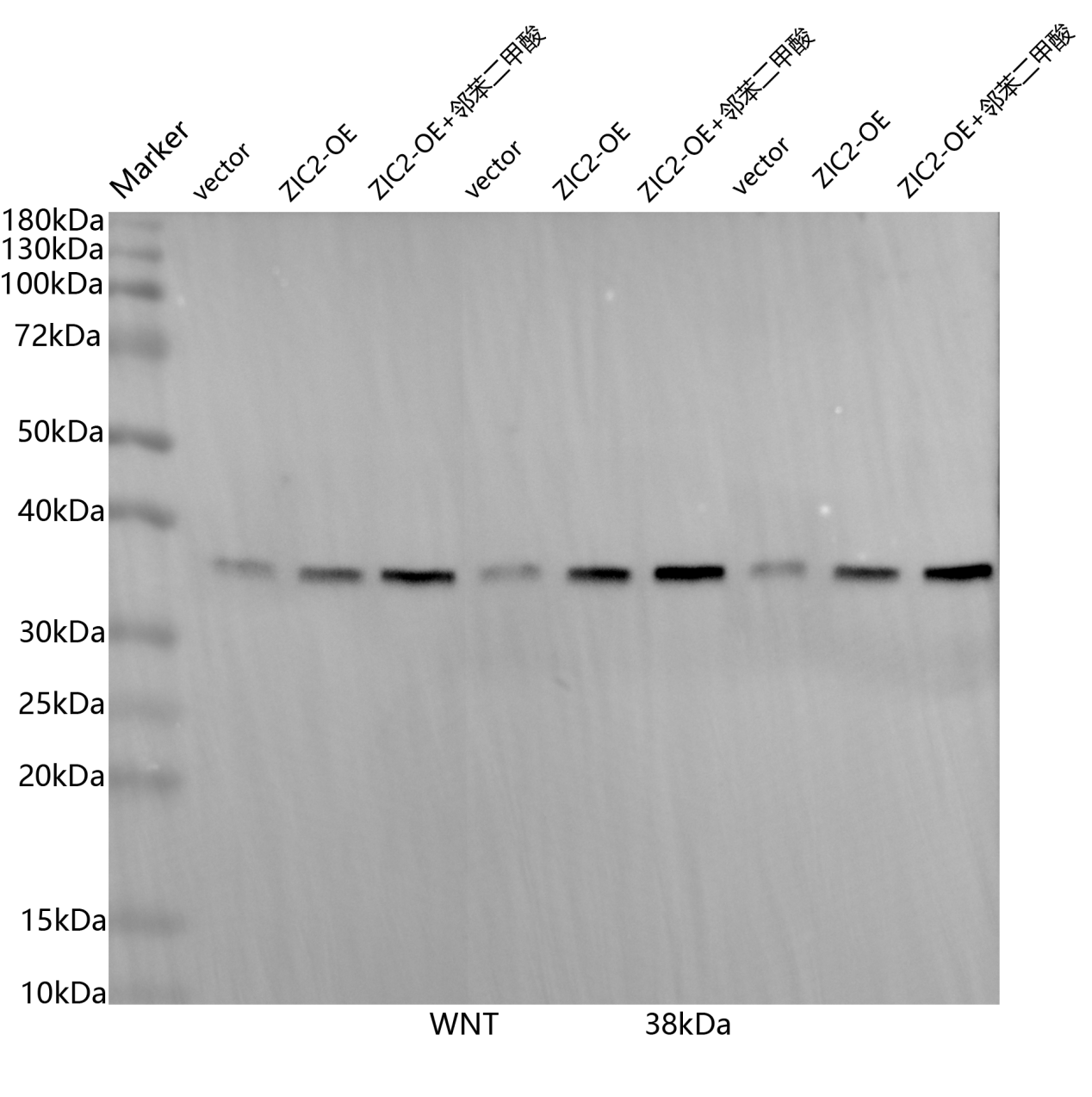


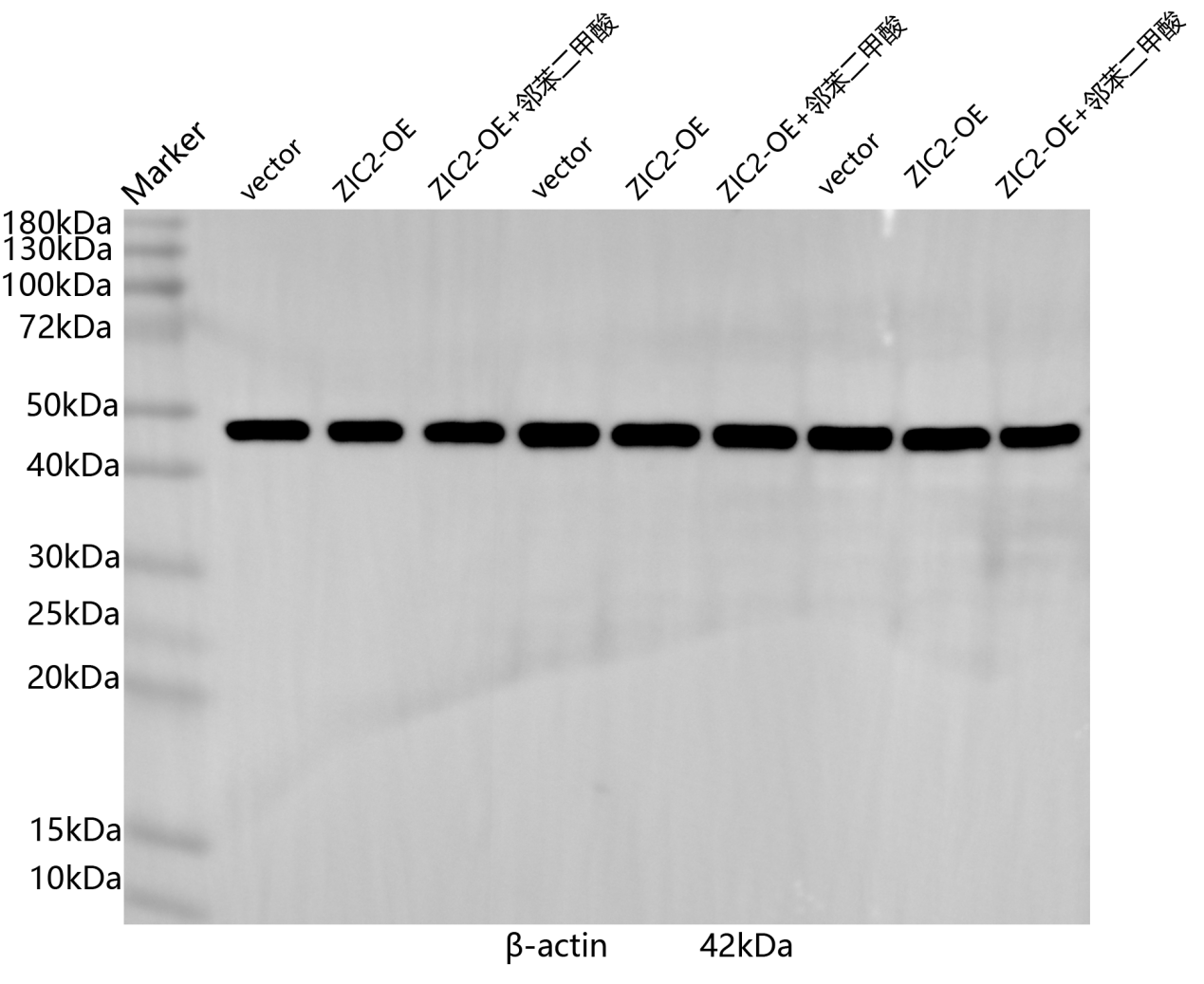


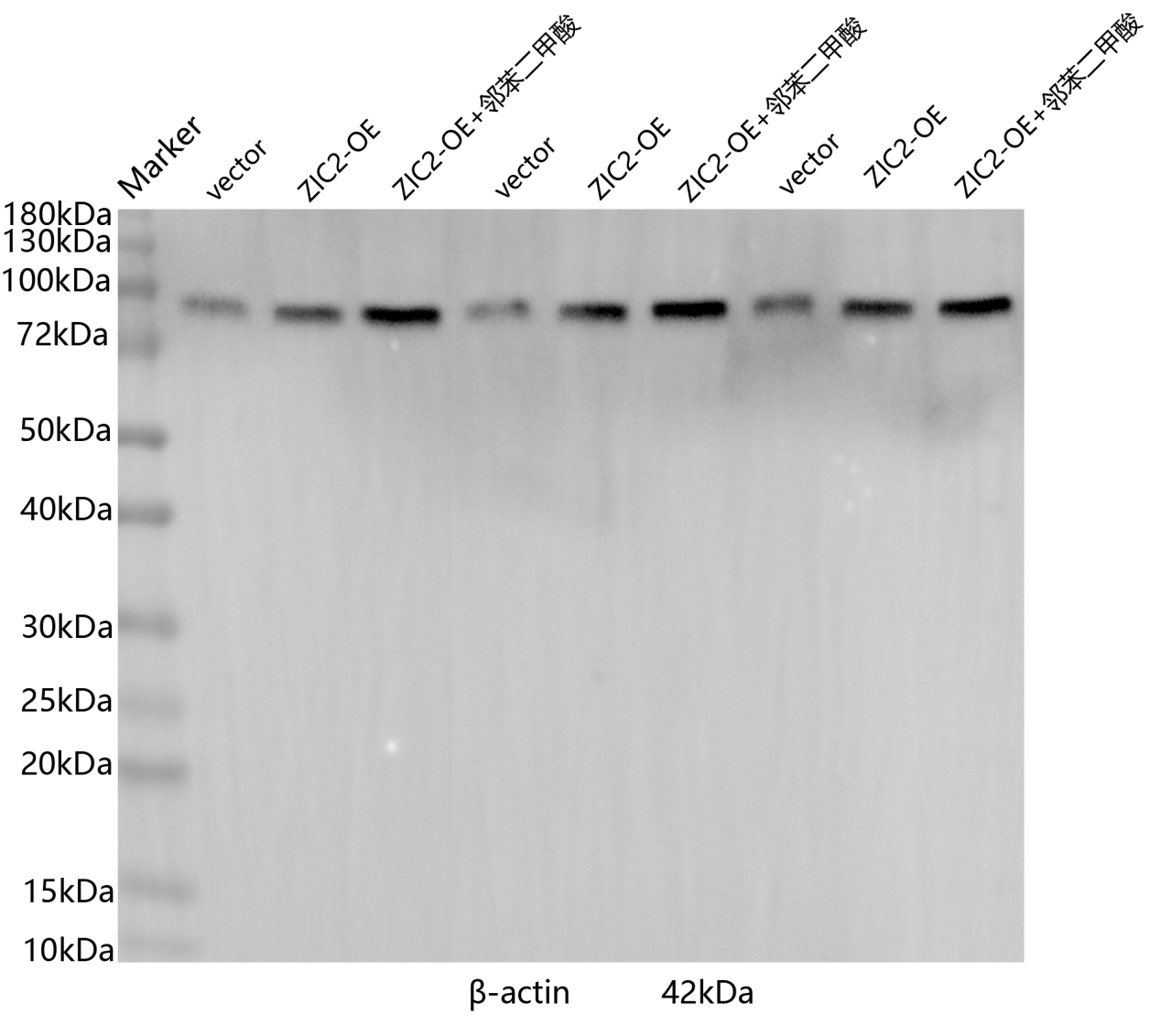

Supplement: Supplementary file 2 [file Table1.docx]
